# An Integrated Perspective of Evolution and Development: From Genes to Function to Ear, Lateral Line and Electroreception

**DOI:** 10.3390/d13080364

**Published:** 2021-08-07

**Authors:** Bernd Fritzsch

**Affiliations:** Department of Biology & Department of Otolaryngology, University of Iowa, Iowa City, IA 52242, USA;

**Keywords:** neurons, brainstem nuclei, hair cells, bHLH genes, *Sox2*, *Eya1*, *Lmx1a/b*

## Abstract

Four sensory systems (vestibular, lateral line, electroreception, auditory) are unique and project exclusively to the brainstem of vertebrates. All sensory neurons depend on a common set of genes (*Eya1*, *Sox2*, *Neurog1*, *Neurod1*) that project to a dorsal nucleus and an intermediate nucleus, which differentiate into the vestibular ear, lateral line and electroreception in vertebrates. In tetrapods, a loss of two sensory systems (lateral line, electroreception) leads to the development of a unique ear and auditory system in amniotes. *Lmx1a/b, Gdf7, Wnt1/3a, BMP4/7* and *Atoh1* define the lateral line, electroreception and auditory nuclei. In contrast, vestibular nuclei depend on *Neurog1/2, Ascl1, Ptf1a* and *Olig3*, among others, to develop an independent origin of the vestibular nuclei. A common origin of hair cells depends on *Eya1, Sox2* and *Atoh1*, which generate the mechanosensory cells. Several proteins define the polarity of hair cells in the ear and lateral line. A unique connection of stereocilia requires CDH23 and PCDH15 for connections and TMC1/2 proteins to perceive mechanosensory input. Electroreception has no polarity, and a different system is used to drive electroreceptors. All hair cells function by excitation via ribbons to activate neurons that innervate the distinct target areas. An integrated perspective is presented to understand the gain and loss of different sensory systems.

## 1. Introduction

Sensory maps depend on the specific sensory modality and the relevant information to be extracted by them. Beyond primary sensory maps, central map formation underlies the integration of various sensory modalities, namely the ear, lateral line and electroreception. The four primary sensory maps of vertebrates have unique features and seemingly use distinct molecular cues, cell cycle exit and activity combinations during development, regeneration and plasticity. The evolution of chordates is comparable with the organization of the dorsal spinal cord and brainstem, which is associated with neurons and hair cells in 71,000 vertebrates. On the other hand, we have limited support for the two chordates associated with the neural crest and placodes, hair cells and central brainstem in 31 species of lancelets and 3100 species of ascidians. Fossils appeared approximately 540 million years ago (Mya), and all major bilaterian phyla presented by 500 Mya [1].

The brainstem of vertebrates is organized into rhombomeres (r0–11) that superficially resemble other chordates, lancelet and ascidians [2–4]. A dorsal part of the brainstem expresses a continuation to the spinal cord in vertebrates [5] which is absent in a true brainstem in other chordates. Partial similarity is found in ‘dorsal root ganglia’ in ascidians that resembles the spinal cord in vertebrates, which is absent in lancelets [2,6,7]. Adding these differences in chordates, gene duplication [8], followed by diversification [9,10], is the basis for the unique brainstem, neurons and hair cells that developed in vertebrates [11]. The unique formation of mechano- and electroreception evolved in four distinct sensory inputs that are partially similar with the lateral line of ascidians [6,12–14], The progression must start with the sensory neurons that connect all neurons with the brainstem and reach out the peripheral sensory hair cells.

**Figure 1.**
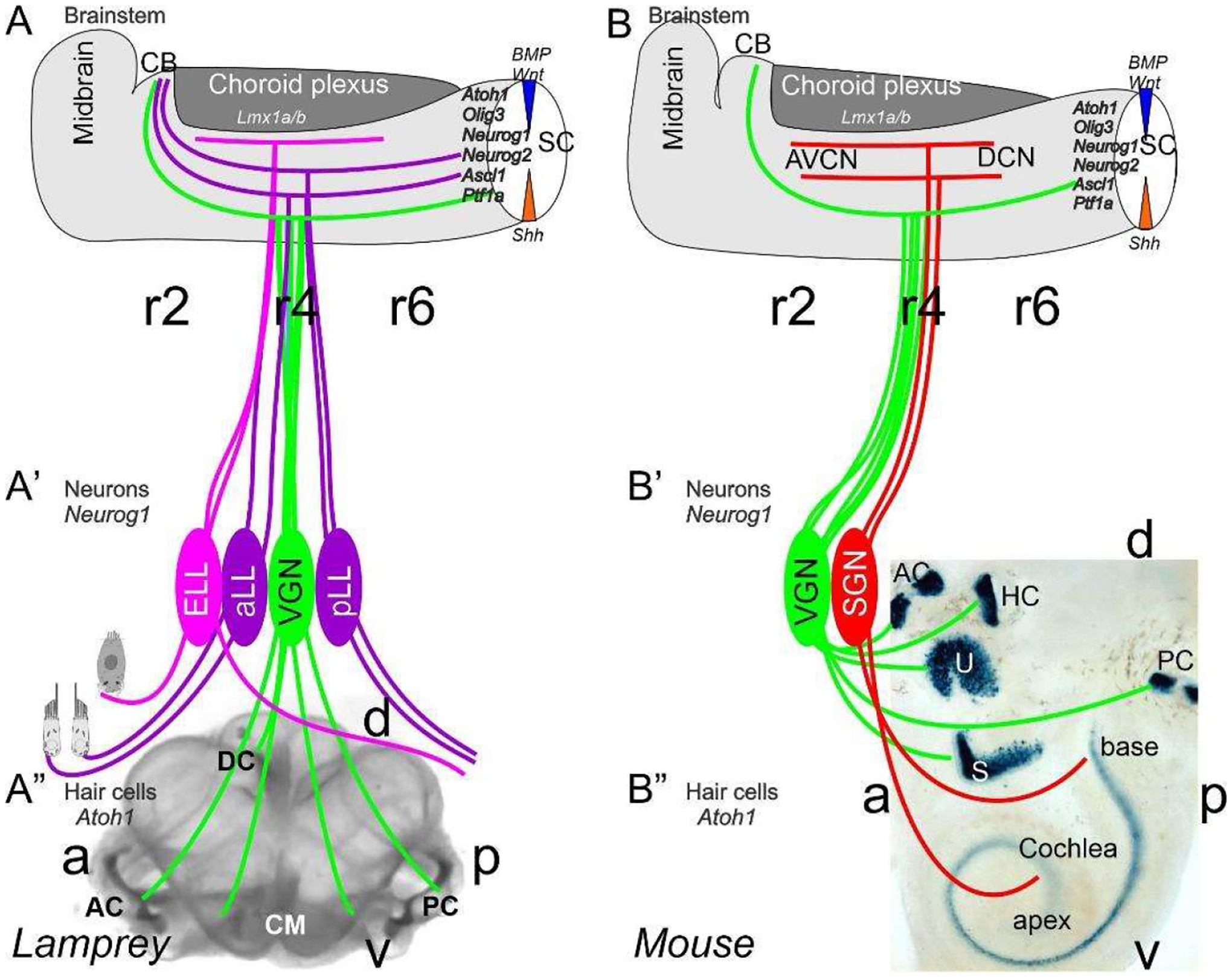
Inner ear, Lateral line and electroreception revealed. Neurons (*Neurog1*; **A′**) form vestibular ganglia (VGN) to reach out 4 hair cell organs in lampreys (**A″**). A separate lateral line (LL) and electroreceptor neurons (ELL) that innervate hair cells project more dorsal in lampreys. Central projection depends on *Atoh1* to receive LL and ELL fibers, whereas several bHLH genes (*Neurog1/2, Olig3, Ascla1, Ptf1a*) receive all VGN (**A**). In the absence of ELL and LL development in amniotes, mammals develop separate spiral ganglion neurons (SGN; **B′**) that extend from the cochlea (**B″**) and end in a topological central projection that depends on *Atoh1* (**B**). The formation of VGNs (*Neurog1*; **B′**) reach the 5 hair cells (**B″**) to extend the distribution of bHLH genes. Note that certain areas are lost or gained which enter central projections near r4. Images are shown by *miR-183* ISH (**A″**) and *Atoh1*-LacZ (**B″**). AC, anterior crista; AVCN, anteroventral cochlear neurons; CB, cerebellum; aLL, pLL, anterior/posterior lateral line neurons; CM, common macula; DC, dorsal crista; DCN, dorsal cochlear neurons; HC, horizontal crista; PC, posterior crista; r2/4/6, rhombomeres; S, saccule; SC, spinal cord; U, utricle. Modified after [11,30,31].

Neurons depend upon Eya1 [15], Sox2 [16], Neurog1 [17] and Neurod1 [18]. In contrast to Neurog1 null mice, which showed a complete loss of neurons [19], Neurod1 null mice showed residual neurons extending centrally to smaller vestibular and cochlear nuclei [20,21] that reached the ear [22,23]. It is worth noting that the lateral line and electroreception are separate for the vertebrate ear that is lost in most tetrapods to generate novel cochlear neurons, the spiral ganglion neurons (Figure 1).

The brainstem is a continuation of the spinal cord (SC; [11,24,25]) that develops into rhombomeres and differentiates into nuclei, namely the vestibular, lateral line and electroreception nuclei in basal vertebrates (Figure 1). Loss of the lateral line and electroreception leads to the development of cochlear nuclei in tetrapods [26,27]. All dorsal expression of the brainstem depends on *Lmx1a/b* [28] and *Gdf7* [29], which drive the choroid plexus (Figure 1). Combined, *Lmx1a/b* and *Gdf7* regulate the formation of *Wnt1/3a, BMP4/7* and *Atoh1*. This formation is likely reduced or absent in *Neurog1/2, Ascl1, Ptf1a* and *Olig3*, among others (Figure 1).

Mechanosensory and electrosensory hair cells (Figure 1) depend on *Eya1, Sox2* and *Atoh1* to initiate the cell cycle and to differentiate into vestibular, cochlear, lateral line and electrosensory hair cells [22,32,33]. Planar cell polarity (PCP) depends on the formation of shifting the central projection of the kinocilium into a lateral position. PCP extends the length of the stereocilia to develop the staircase of tip links of the vestibular, cochlear and lateral line hair cells [34–36]. The next step involves the development of the tip links to allow the connections between *CDH23* and *PCDH15* to open up the channel to form a mechanosensory hair cell [37,38], with opposing polarity in most of the ear and lateral line [34,39–41]. *TMC1/2* provides a major function that seems to interact with additional channel proteins (*TMHS, TMIE*), forming a complex interaction [37,42–44]. In contrast, while the electroreception forms next to lateral line hair cells [22,23,45], these hair cells lack any polarity organization, and certain ampullary hair cells are dependent on *Ca*_*v*_*1.3* [46].

This review will compare the three neurosensory components that form the neurons which, on the one hand, connect to the brainstem for input, and, on the other hand, receive the hair cells for sensory input. Gene regulation of neurons, central nuclei and hair cells is driven by gene duplication and diversifies after chordates diverge from vertebrates [10], leading to the gain and loss of three sensory systems (lateral line, electroreception, auditory). Gene regulation explains the diversification of the vestibular system from three hair cells up to nine hair cell populations, including the cochlea of mammals [3,47],

## 2. Neurons Depend upon *Eya1, Sox2, Neurog1* and *Neurod1*

**Figure 2.**
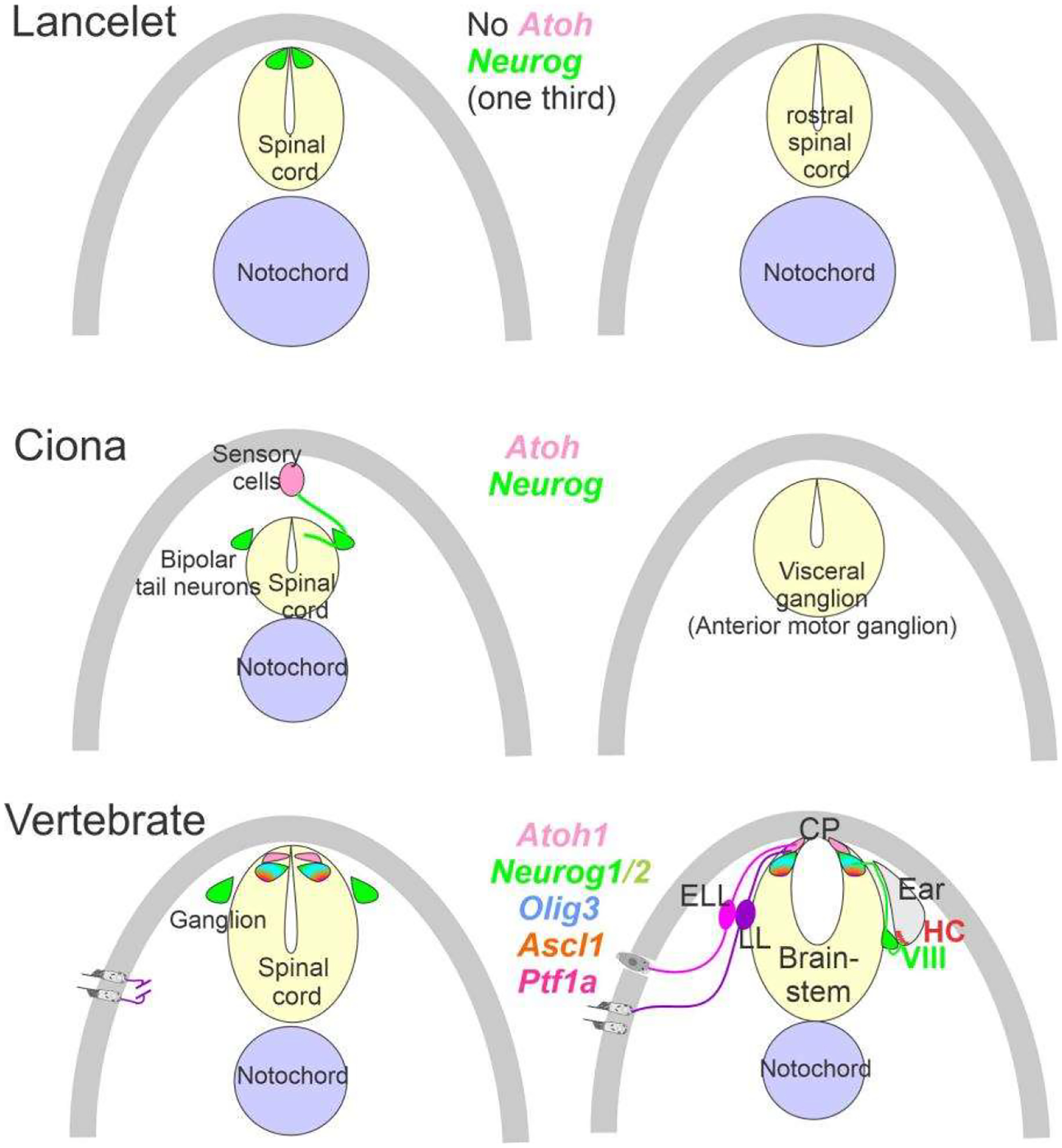
Neurons require *Neurog* expression. Lancelets have a limited description of bHLH genes that are characterized in the more caudal spinal cord, which is positive for *Neurog*. Note that the lancelet has no *Atoh* bHLH gene. Ciona has at least 6 bHLH genes expressed in sensory cells that are innervated by bipolar tail neurons which extend to reach the visceral ganglion for interactions. *Atoh* and *Neurog* genes are described in Ciona associated with the spinal cord. Vertebrates have dorsal root ganglia that depend on *Neurog1/2*, which is also expressed in *Atoh1* and *Neurog1* of the spinal cord. The brainstem is innervated by electroreceptor (ELL) and lateral line fibers (LL) that extend to innervate migration populations of LL and some ELL). The ear is unique in vertebrates, which give rise to the VIII ganglia that innervate more ventral nuclei compared to LL and ELL projections to reach *Atoh1*. CP, choroid plexus. Modified after [2,7,12,23,24].

The ear, lateral line and electroreception neurons depend on genes that, collectively, define their development. Upstream of bHLH genes, which initiate the proliferation of neurons, is the expression of *Eya1*, which interacts with *Brg1* to initiate pro-neurosensory development [15,48,49]. In the absence of *Eya1*, there is no neuronal development that allows ear formation, and neither neurons nor hair cells differentiate [15]. Evolving neurons start in the lancelet, which lack dorsal root ganglia. The dorsal root ganglia show partial expression of *Neurog* inside the spinal cord (Figure 2), which lacks an *Atoh* gene [50,51]. In contrast, at least a smaller set of bHLH genes are partially characterized in the developing ascidian, *Ciona* [52], which have at least six bHLH genes driving neuron development: *Ptf1a, Tcf3, Atoh, Ascl* and *Neurog* [7,12]. A detailed serial section analysis shows the innervation of sensory cells (*Atoh*) from fibers of the neurons (bipolar tail neurons; Figure 2) that can trace to reach the anterior motor ganglion [13]. Neither the full expression of *Eya* nor *Sox2* outside the neural plate are unclear in the lancelet and tunicates [2,52].

**Figure 3.**
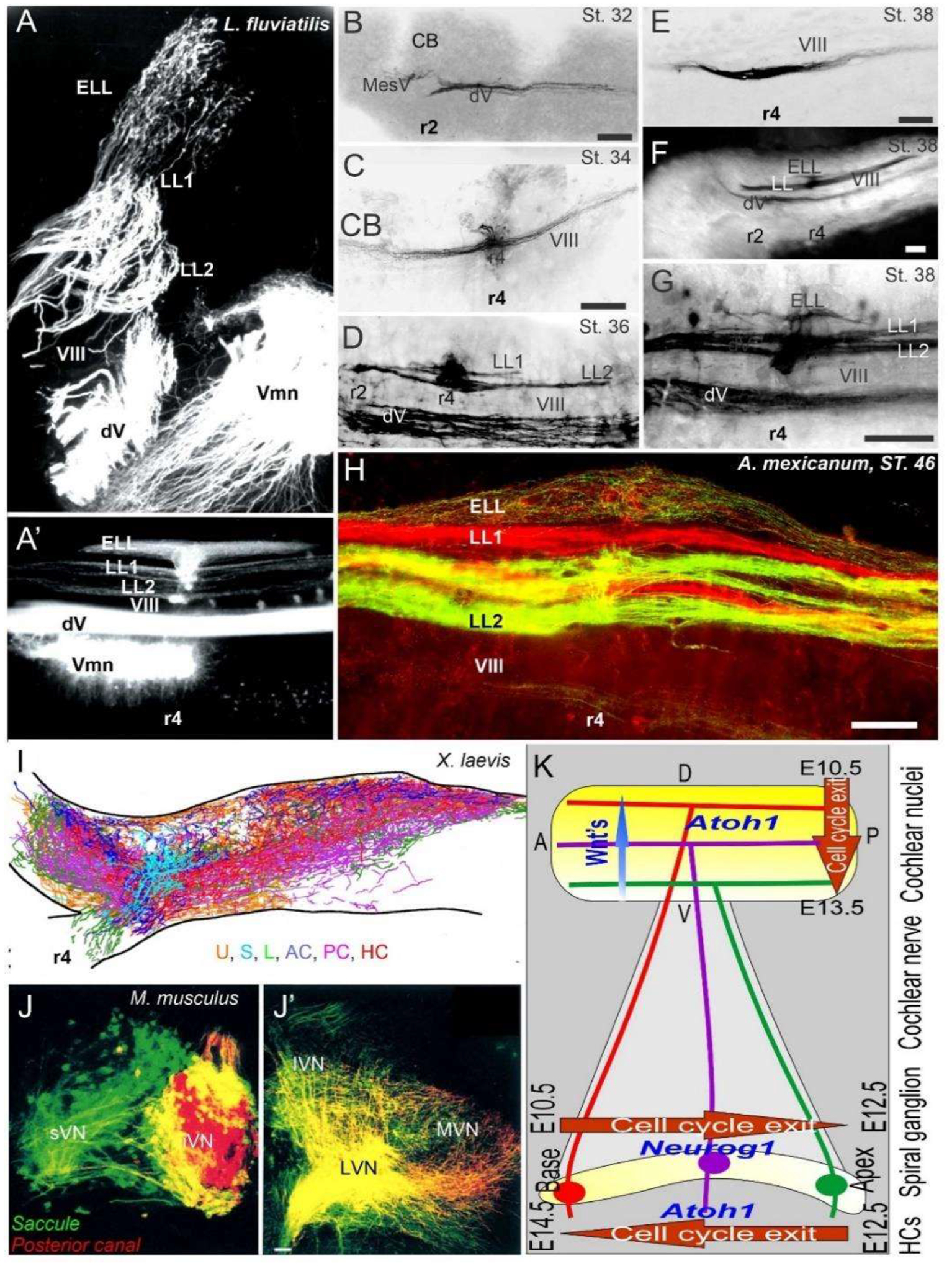
Central projections form afferents to distinct innervation. The lateral line of 2 or more branches form, whereas electroreception receives the short dorsal projection in lampreys (**A,A′**) and salamanders (**B-H**). Vestibular projection forms after the trigeminal central projection, followed by the lateral line and electroreception (**B-H**). Central projection in a frog (**I**) and mammal (**J,J′**) show the incomplete distribution of distinct neurons (**J**) that overlap and incompletely segregate the vestibular projection (**I,J′**). Spiral ganglia (**K**) proliferate neurons in a base to apex progression (E10.5–12.5) that reach the central projection to form a topology from dorsal to ventral cochlear nuclei (E10.5–13.5), depending on Wnt expression. Later, hair cells proliferate from apex to base (E12.5–14.5) that reach the afferents. AC, anterior crista; dV, trigeminal afferents; ELL, electroreception; HC, horizontal crista; LL1/2; lateral line; L, lagena; LVN, lateral vestibular nuclei; IVN, inferior vestibular nuclei; iVN, inferior vestibular neurons; MVN, medial vestibular nuclei; PC, posterior crista; S, saccule; sVN, superior vestibular neurons; U, utricle; Vmn, trigeminal motoneurons; VIII, vestibular projections. Modified after [3,23,67,123].

A crucial next step is the initiation of *Sox2*, which is needed to upregulate *Neurog1* [53–55]. In fact, *Sox2* delays certain neuron development in bony fish [56], and in the presence of *Sox2* is unclear the sequence of gene regulation in the lamprey and hagfish [57]. There is a distinct effect of the loss of early genes in the vestibular ganglion, which initially differentiates in the absence of *Sox2* and *Neurog1* (Figures 1 and 2) and does not develop in the auditory neurons [16]. A loss of all auditory neurons, and partial loss of vestibular neurons, are known for *Pax2* [58], *Gata3* [59], *Lmx1a/b* [28], *Fgfr2* [60], *Shh* [61] and *Dicer* [62]. Partial loss of some vestibular neurons are known for *Fgf10* [63] and *Foxg1* [64,65], indicating a limited loss of sensory hair cells and/or neurons. Unfortunately, the details of the lateral line and electroreception (Figures 1–3) are not as fully genetically characterized [22,23,27,33]. The lateral line and electroreception likely depend on neuronal development (Figures 1 and 2), including the development of spinal ganglia neurons [66] and trigeminal neurons [67–69]. A separate placode is derived from neurons that develop from *Neurog1* in mammals [68,70]. In birds, this placode is driven by *Neurog1* [71,72]. Furthermore, separate amniotic paratympanic placodal neurons innervate separate hair cells that partially integrate into the central vestibular projection [72].

In addition to directly initiating the formation of neurons by *Eya1, Sox2, Pax2* and *Neurog1/2*, another set of genes are regulated to differentiate into *Neurod1* [18,20,21,71,73], followed by *Isl1, Foxg1, Pou4f1* and *Phox2b* [71,74–76], which interact with *Shh, BMPs* and *Wnts* to define neurons [77,78]. Regional regulation of the distinct vestibular, lateral line, electroreception and auditory neurons are sorted out by downstream genes regulating the distinct innervation. For example, the expression of *Calbindin, Calretinin, Pou4f1* and *Peripherin* is required to sort out the innervation from the inner and outer hair cells [79–82]. In *Sox10* null mice, an interaction showed disorganized cochlear neurons, whereas the development of vestibular neurons was near normal [83]. This interaction is consistent with the loss of *Erb2* of nearly all cochlear neurons, as well as reduced vestibular neurons [84]. The concept of having multiple sources of neurons from the placode and neural crest is likely due to a misinterpretation [3,83,85–87].

Downstream of gene development, the expression of *TrkB (Ntrk2)* and *TrkC (Ntrk3)* has a reduction and loss in vestibular and cochlear neurons. Vestibular neurons are mostly dependent on *TrkB* [88,89] whereas the cochlear neurons are mostly dependent on *TrkC* [90,91]. Loss of both neurotrophin receptors causes the early loss of all neurons [92–94]. Limited expression is characterized in some ascidians which are unknown in the lancelet [1]. The comparable expression of the lateral line and electroreception are unclear due to the multiplication of neurotrophins in bony fish [95,96].

The proliferation of neurons and hair cells depend on *MycN* [97,98], which drives the division of the Gl, S and G2 phases with a set of genes that interactions with cell cycle regulation [53,99–101]. Detailed characterization and proliferation have been described in the ear and brainstem, clarifying cell cycle progression in mice and rats [102–104]. *Sox2* and *Neurog1* are in negative feedback, which allows proliferation and initiates differentiation. This differentiation interacts with retinoblastoma *(Rb), Hes/Hey* and *IDs* to regulate the cyclin-dependent kinases (*CDKs*), cross-react with e-proteins and define whether a cell cycle is progressing [98,100,105,106]. In the end, continuation depends on either knocking out *Rb* to continue proliferation or upregulating of *Sox2* to jumpstart proliferation [107,108].

In various vertebra, the central projection has been described to show the projection of the vestibular, lateral line, electroreception, and cochlea [3,67,87,109–111]. Three sets of central projections are known in vertebrates that develop a loss of the lateral line, electroreception and added cochlear nuclei [23,26,112]. For electroreception, these central projections always have a single set of an anterior ganglia (Figures 1 and 3) that adds variably the electroreception in bony fish [27,113]. Lateral line neurons (Figures 1–3) can be split into an anterior and posterior branch that diversify the neuromasts to innervate all lateral line hair cells (Figure 3; [114–116]). Vestibular neurons have two neuron populations in hagfish [57], while lampreys and jawed vertebrates have a single vestibular ganglion [111,117,118]. At least 4–5 distinct innervations are described in lampreys [119,120], whereas most gnathostomes have at least five and up to nine branches of vestibular and auditory connections (Figures 1 and 3): three canal cristae, utricle, saccule, lagena, basilar papilla, amphibian papilla and neglecta [121,122]. Branches of discrete neurons are known for an anterior and a posterior (superior) nucleus that innervates two canal cristae (anterior and horizontal cristae), the utricle and part of the saccule (Figure 3). The remaining part of the utricle provides a posterior canal and the branch of the saccule (Figures 1 and 3) in mammals [123]. The development of central projections follows a simple layout. First, the trigeminal and epibranchial neurons develop. Then, central projection follows. Subsequently, vestibular, lateral line and electroception develop, if present (Figure 3; [3,124]). Different developmental patterns exist in neuronal proliferation: nearly all neurons continue proliferation for a long time or lifetime, whereas mammals have an early production of neurons that ends proliferation very early [67,125,126]. The topology of peripheral neurons of the vestibular, lateral line and electroreceptors is unclear, suggesting an overlap with an incomplete segregation of neurons that is well known for the vestibular neurons (Figure 3 [123]).

A long-term proliferation of the vestibular, lateral line and electroreception is followed by a delayed formation of cochlear neurons, the spiral ganglia neurons (SGN), which follow vestibular neurons in mammals (vestibular neurons: E9–11; SGN: E10–12 [125,127]). A unique topological development is known among mammals [128], first showing the basal turn neurons (Figure 3), which reach the anteroventral, posteroventral, and dorsal cochlear nuclei (AVCN, PVCN, DCN). The development of these neurons is followed, with delay, by the apical neurons [67,87,110,129]. Interestingly, there are central projections that can form independently to reach the formation of cochlear nuclei [130]. In the absence of target hair cell development [92,131], cochlear neurons develop and largely proliferate prior to cochlear nuclei and cochlear hair cells (Figure 3). Central cochlea require the expression of *Neurod1, Wnts, Fzd, Npr2* and *Ephrins* for targeted central projections [21,129,132,133].

In contrast to the topology of the cochlear nuclei [11,128], the central vestibular neurons have an incomplete central segregation (Figure 3) that shows both segregation and overlap from different vestibular neurons [3,123,134]. Lateral line central projections can be segregated in certain vertebrates but show an overlap in other vertebrates [3,23]. For electroreception, multiple central topological projections in certain bony fish [27,135] show an overlap in lampreys and salamanders (Figure 3 [23,109]). The vestibular, lateral line, electroreception and cochlea independently reach hair cells that form prior to neurons [23,136], consistent with the same pattern of neurons that develop first, followed by the central axon to the brainstem, and later followed by the hair cell innervation [3,109,134,137]. This is obvious in cases where hair cells are not formed, such as in *Atoh1* null mice, which show a near-normal central projection [131,138]. A similar central projection forms after the loss of hair cells in *Pou4f1* null mice [139]. Loss of formation of a specific set of hair cells is demonstrated in the posterior canal that projects normally, despite the absence of *Fgf10* [63], which degenerates later.

In summary, the neurons of the ear, lateral line and electroreception are generated by a set of genes that act downstream of *Neurog1* to initiate the cell cycle. Neurons develop independently of central axons and reach innervate the hair cells shortly after proliferation. Segregation of central projections can be topologically organized in the auditory central projection of most tetrapods, and present two lateral line neurons that segregated in many vertebrates. Some central topology found in some, but not all, lateral line and electroreceptors, show an incomplete segregation for the vestibular neurons.

## 3. The Brainstem Is Transformed from the Spinal Cord

The spinal cord and rhombomeres (r0–11) of the brainstem [140,141] are basically identical in terms of the distribution of overall gene expression [24,25]. The distribution of gene expression in the spinal cord and rhombomeres differentiates into a unique population of r0–7 [142–145]. The earliest genes— *Gemini (Gmnn), Zic* and *Foxd4* [146–148]—define the neural ectoderm, which cooperates with *Smarca/Brg*-related genes to induce neural ectoderm. Certain interactions can become more complicated and can, for example, be downstream from *Zic1* by *Wnt1* and cooperate with *Fgf, Noggin/Chordin* and *Nodal*, which counteract with *BMPs* while *Dkk/Cerberus* counteracts *Wnt*. Interestingly enough, certain aspects of *Wnt* are independently regulated from *Wnt3a*, defining more variations among the large family of *Wnts* [149,150]. A major role for the invaginating of neuroectoderm depends on *Shh* and *Gli* to induce ventral formation, which counteracts with *BMPs* and *Wnts* to define the dorsal part of the brainstem and induces the motoneurons [4,151,152].

**Figure 4.**
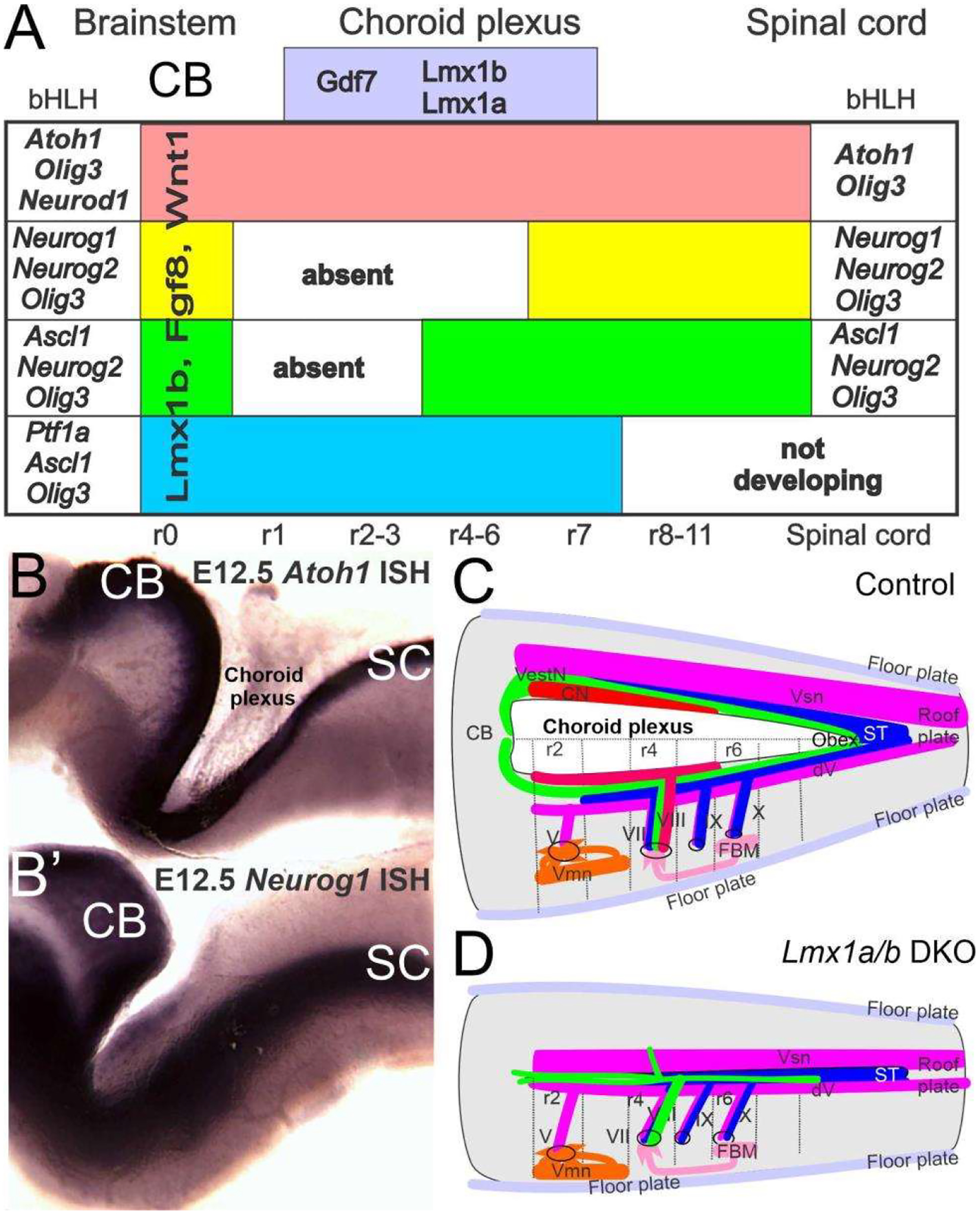
The brainstem depends on *Lmx1a/b, Gdf7* and bHLH genes. The choroid plexus is unique, forming in the brainstem. The choroid plexus depends on *Lmx1a/b* and *Gdf7* (**A**), and is replaced for the roof plate in the spinal cord (**C,D**). Downstream are bHLH genes that have been identified in the *Atoh1* (**A,B**), *Neurog1* (**A,B′**), *Neurog2, Neurod1, Ascl1, Olig3* and *Ptf1a*. Certain expression is unique for the vestibular and auditory nuclei: *Ptf1a* is a duplication of ventral genes that are replaced by more rostral genes (*Neurog1, Ascl1). Lmx1b, Fgf8* and *Wnt1* are common cerebellums (CB) of r0. In the absence of *Lmx1a/b* and choroid plexus, no cochlear nuclei form and vestibular, trigeminal and solitary tract interact across the roof plate (**C,D**). dV, trigeminal fibers; FBM, facial branchial motoneurons; ST, solitary tract; V, VII, VIII, IX, X, afferent fibers; Vmn, trigeminal motoneurons; Vsm, trigeminal nucleus. Modified after [28,82,157].

Recent work has shown that a unique formation of the choroid plexus in the brainstem depends, at least, on two genes: *Lmx1a/b* and *Gfp7* [29,153,154]. In the absence of *Lmx1a/b* double-null mice, the choroid plexus disappears (Figure 4), transforming the dorsal part of the brainstem and cerebellum into a continuation from spinal cord to the midbrain [11,28].

Gene expression of *Eya1* [74,155], followed by *Sox2* [15,53,54], is needed to upregulate proneuronal formation. In addition, a set of bHLH genes [5,24,25] is required to initiate the formation of neurons. Only two bHLH genes, *Atoh1* and *Olig3*, are expressed throughout the spinal cord and brainstem [5,25,156] that is diversified in the more rostral part of the brainstem into the cerebellum and auditory nuclei [157]. The formation of all neurons that depend on *Atoh1/Oligi* shows complete loss of all *Atoh1* expression genes [158].This formation has been demonstrated using *Wnt1-cre* upstream of *Atoh1*, leaving only the choroid plexus in *Atoh1* null genes [130,156]. In contrast, some AtoM-positive cells develop in *Olig3* null mice that have changed the definition of the effect without *Olig3* [145,159]. Loss of *Gdf7* [29] and *Lmx1a/b* double-null mice [154] abolishes *Atoh1* expression, *Olig3* remains that may or may not expressed in *Gdf7/Lmx1a/b* mice (Figure 4).

A complex interaction is generated by feedback loops. J Johnson showed the cross-repression of *Atoh1-Neurog1* in a reciprocal interaction to sharpen the boundaries of *Atoh1* and *Neurog1/2* in the spinal cord [24]. Different expression levels define (from roof plate) *Atoh1, Neurog1/2, Ascl1* and *Ptf1a*. In addition, roof plate is regulated by *Gdf7* and *Lmx1a/b* to follow a gradient of high levels of *BMP* and *Wnt. Atoh1-Neurog1/2* is not only repressed, but is also expanded by *Ascl1*. This expansion defines most ventral fate and expresses *Neurog1/2* adjacent to the same expression. *Ptf1a* is, again, a repression interaction with *Ascl1* and defines a subdomain in the spinal cord [24] and brainstem.

In comparison to the spinal cord, certain gains and losses of domains are clear. For example, another unique step is driven by an apparent *Ptf1a* duplication in the brainstem [25], which results in *Ptfila* null mice, a specification of more dorsal into a different state of r0–7 [142,143,145]. More complex loss of *Neurog1/2* in r1–6 and part of *Ascl1* in r1–3 replaces the more dorsal expression of *Ptf1a* [25,143]. A more rostral reduction of these two domains requires additional research to explain the distinct effects of *Ptf1a* null mice [142,143]. In essence, the spinal cord has six identical domains (A1–3, B1–3) that differ from the rhombencephalon, showing the differential gains and losses of two domains (dA2, dA3). The spinal cord has the ability to develop two additional domains, for a total of eight domains, (A1–4, B1–4) which highlights the gains and loss of selective bHLH genes [25].

In addition to this cross-interaction, the spinal cord is further expanded by another bHLH set of genes, the *Hes/Her* genes [53,160] and the *ID* genes [9,99,161]. Starting with *Sox2* expression, the neurosensory precursor cells are self-renewing and are driven by the *Hes, ID* and *Myc* genes to enhance proliferation [105]. The expansion changes by an oscillation to interact with *Hes/Ascl1*, for example. It is important to understand that the *Notch* interaction allows neurons to differentiate while precursors remain as neural stem cells. In the dorsal part of the spinal cord and brainstem, the genes interact with *Atoh1, Neurog1/2, Olig3, Ascl1* and *Ptf1a* among proneuronal bHLH genes. Diversity is driven by distinct ways to generate astrocytes. In contrast to a downregulation of *Hes/Id/Myc, Sox2* is essential for neurosensory cell formation to differentiate in astrocytes that remain in *Hes, Id* and *Sox9*, among others [54]. In contrast, oligodendrocytes are equally downregulated, such as in neuronal differentiating cells through upregulation by *Olig1/2* and *Sox10*.

*Atoh1, Neurog1/2, Olig3, Neurod1* and *Ptf1a*, among others [145,157], define the cerebellum (Figure 4). A delayed expression of *Neurod1* adds to the interaction by providing negative feedback for the cerebellum of at least *Atoh1* [157,162], which expands along the auditory nuclei for feedback. Likewise, identical expression in the hindbrain shows a near-equal expression of *Atoh1* (rostral) and *Neurod1* (caudal). However, in the adult system, a different level of *Atoh1*, which shows a much higher level of expression in the auditory nuclei, supposedly counteracts with *Neurod1* out of two nuclei, particularly the dorsal cochlear nucleus [157]. In summary, the cerebellum depends on multiple genes (*Olig3, Atoh1, Neurod1, Ptf1a*, among others), and the exact genes are unclear in lamprey and hagfish [145,157,163].

*Lmx1a/b, Fgf8* and *Wnt1* delineate the cerebellum [141,152,153]. In the absence of *Lmx1a/b*, fibers branch to reach unusual central projections of vestibular fibers that receive fibers from the trigeminal and the solitary tract, crossing the nearly closed roof plate (Figure 4). Consistent projections receive the innervation from the vestibular neurons or can expand to reach lateral line fibers in vertebrates (Figures 1 and 4). Neither the electroreception nor the cochlear fibers expand to reach the cerebellum that do not expand beyond r2 (Figures 1 and 4). Certain changes in the auditory fibers can transiently trace to reach the cerebellum in certain mutations [129,164] that never directly reach the electroreceptors [27,135].

Higher projection to the midbrain and telencephalon is known among auditory, vestibular, lateral line and electrorections. However, this topic is out of the scope of this review [3,26,27].

In summary, the four dorsal nuclei depend on bHLH genes that define a complex interaction by the gain and loss of other bHLH genes that cross-correlate, for example, *Atoh1* and *Neurod1* in the cerebellum and auditory nuclei. Without *Lmx1a/b*, there is a loss of the choroid plexus, as well as the loss of *Atoh1* and likely other more dorsal brainstem genes (*Neurog1*, *Neurog2, Neurod1, Olig3, Ascl1* and *Ptf1a)*.

## 4. Hair Cells Depend on *Eya1, Sox2* and *Atoh1*

**Figure 5.**
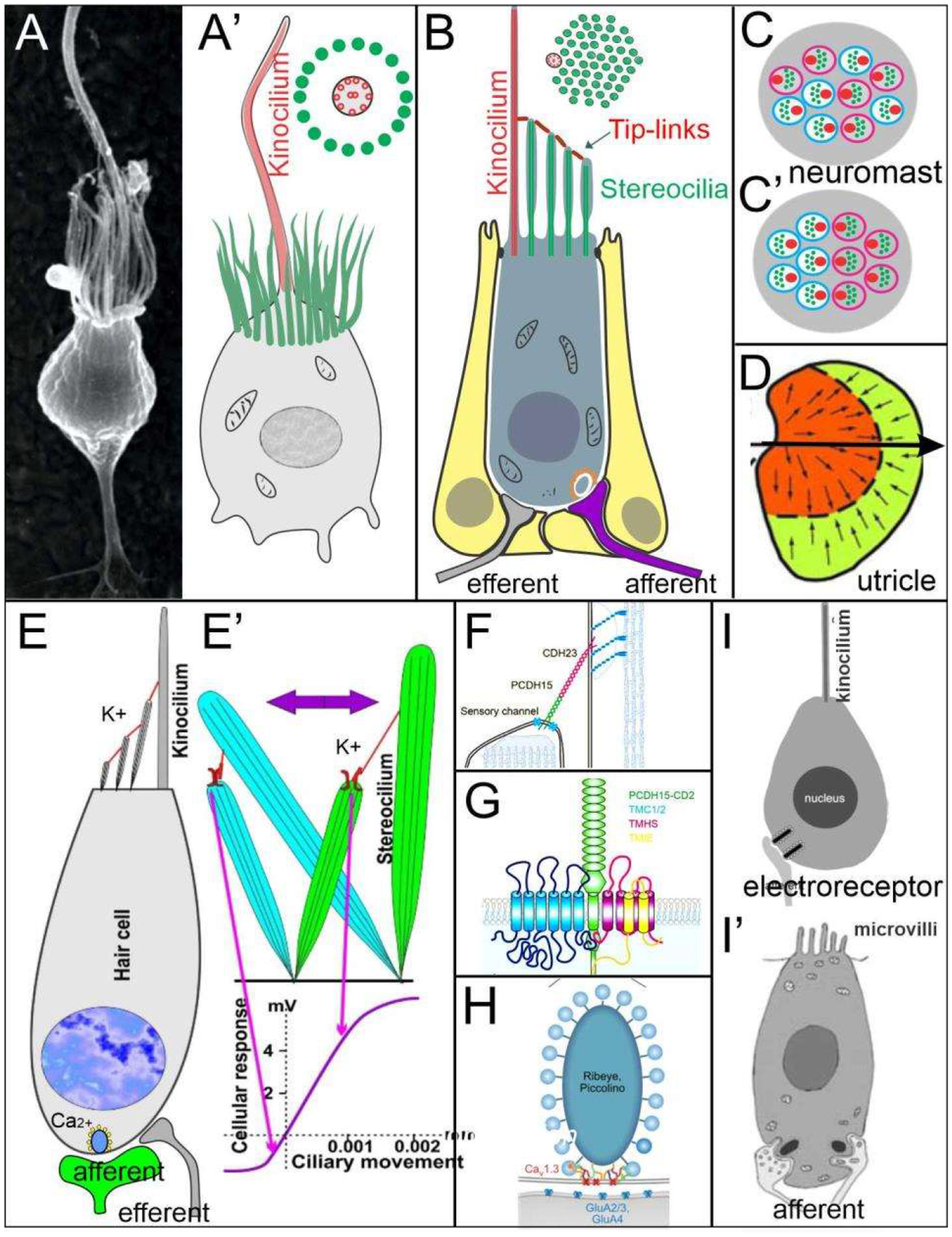
Mechanosensory hair cells evolve from single-cell organisms. Choanoflagellate (**A**,**A**′) are the basis of animals that evolved from a kinocilium surrounded by microvilli into an asymmetric staircase of mechanosensory hair cells (**B**,**C**,**E**) that forms the mechanoelectrical transduction channels (MET) of the lateral line (**B**,**C**,**C′**) and vestibular hair cells (**E**). The lateral line (**C**,**C**′) and some vestibular hair cells (**D**) are bipolar, whereas canal cristae and most auditory organs are polarized in 1 direction. Tip links depend on CDH23 and PCDH15 (**F**) that interact with *Tmc* and others (**G**) to open up the channel (**E′**) to allow K+ entrance. Ca2+ interactions with t ribbons to allow the release of glutamate (**E**,**H**). Electroreceptors are unpolarized and resemble Choanoflagellate that either show microvilli (**I**′) or only a central kinocilium (**I**). Modified after [22,37,47,196,211,212].

Mechanosensory hair cells are shared among the vestibular, cochlear, lateral line, electroreceptor and Merkel cells, a unique late addition to trigeminal sensory information [3,11,135,165]. Hair cells and Merkel cells depend on *Atoh1* for differentiation [166,167]. Evidence suggests that hair cells evolved from single-cell organisms, called choanoflagellates [32,47], which transformed a single kinocilium surrounded by villi (Figure 5) into distinct hair cells, the mechano- and electrosensory hair cells. In addition to vestibular hair cells, the inner ear forms a set of 3–9 patches of hair cells, including the cochlear hair cells (Figure 1; [117,122]). Lateral line hair cells distribute from small clusters of hair cells, referred to as neuromasts (Figure 5), to form a large set of hair cells in sharks [23,115]. Electroreception can subdivide into the ampullary organs of basic vertebrates, various additional bony fish have evolved several sets of ‘electroreceptors’ (Figure 5; [22,27,46]).

The vestibular ear requires a set of transcription genes to initiate the placode formation, starting with *Foxi3* [168] and *Fgf3/10* [63,169,170]. Downstream are *Eya1/Six1* [49,171], *Pax2/8* [58,172], *Shh* [78,173], *BMPs* [174,175] and *Wnt’s* [176–178] to form the otocyst, among other necessary genes [179], where they interact to define the dorso/ventral, anterior/posterior and lateral/medial divisions to develop the otocyst [180,181]. Further downstream is the expression for *Sox2* upregulation [16,182]. *Sox2* upregulation sets up the differentiation into hair cells, which depends on the cross-interaction of *Atoh1* with *Neurod1* [21,183], *Pou4f3* [139,184,185], *Gfi1* [184,186], *Srrm/Rest* [187,188] and *Barhl1* [189,190], among others, which differ in efferent and afferent innervation [191–194].

Vestibular hair cells form maculae for gravistatic reception and canal cristae for angular receptions [47,195,196]. Polarity depends on function, but the distribution of hair cells differs. Only maculae have opposing maculae (Figure 5), whereas canal cristae are uniform in their polarity [117,191,196]. Canal cristae are also present in most auditory hair cells [122,197]. Sensory hair cells form Type I and Type II hair cells in amniotes have a common organization. All vertebrate hair cells have stereocilia organized in a staircase pattern, displaying distinct apical polarities for stimuli to open mechanoelectrical transduction channels (METs) by tip links using PCDH15 and CDH23 (Figure 5), permitting endolymphatic potassium to enter the HCs and change their resting potential [37,197,198]. The mammalian mechanosensory channel is, in part, formed by the transmembrane proteins *Tmc1* and *Tmc2* [38,199]. Other interactions are known, but these interactions require additional work for the MET formation (Figure 5). A unique formation of vertebrate hair cells is found in the *Tmc1/2* single gene in cyclostomes [43]. *Tmc1/2* is separated from the closely related gene, *Tmc3*. However, the function of *Tmc3* is unclear nearly all animals, including basic animals, for which there is no information regarding its function.

Planar cell polarity (PCP) genes depend on *Frizzled, Prickle, Disheveled, Van Gogh, Diego* and *Flamingo* for normal development [200,201]. Polarization depends on *Emx2* [41], which eliminates the contralateral organization in the utricle by converting it into a single polarity [202,203]. In addition, retinoic acid (RA) sets up various gradients [204]. Saccule and lagena have a different polarity. Instead of polarizing each other again in the utricle, they flip to organize in the saccule and lagena [191]. A distinct pattern of the utricle and saccule have a separate innervation from the cerebellum to reach one polarity (Figure 5D) and receive a descending branch of the caudal vestibular neurons [21,134,205] to end up in a different innervation (Figure 5D).

The functional unit of the lateral line system is the neuromast, which physically couples hair cells to the surrounding medium [206]. Within a neuromast, the hair cells are organized in two opposing polarities that are either randomly distributed within a neuromast or occur in a regularized counter-organization (Figure 5). The transduction from the mechanical stimulus requires an eccentric kinocilium and shorter stereocilia [207]. The absence of *Tmc1, Tmc2* or TMIE disrupt stereocilia development [208]. It seems possible that the neurons giving rise to the two afferents, and possibly also the two opposing hair cell populations, are separated by different birthdates in teleosts [124,209]. In zebrafish, it was further shown that, while early-born afferent neurons connect hair cells to the Mauthner cell, those occurring later only project to the central nucleus [210].

The opposing polarity of hair cells and their selective innervation by afferent nerves is determined through the combined action of transcription factor *Emx2* [40,41,213–215]. Ectopic expression of *Emx2* drives all hair cells to organize their kinocilia in a caudal position, while broadly activating the *Notch* pathway results in the inhibition of *Emx2* expression. Thus, all kinocilia are positioned rostrally [40,116,215]. It appears that a bistable situation then determines of *Emx2* in the rostral sibling through *Notch*-mediated lateral inhibition, which then determines the caudal position for the kinocilium of the rostral sibling and the emergence of the opposing polarity [23].

Auditory hair cells are unipolar in mammals and depend on *Vangl2*, *Dvl1*, *Celsr1* and *Gal2* from the PCP pathway [35,216]. *Emx2* and *Jag1* are both needed for the development of OHCs, which increases the IHC [41,217,218]. Electroreceptors show no polarity in either single kinocilium or multiple microvilli [22] which use nonmechanical sensation [46,219]. Efferents have been found in vertebrates, and vertebrates that receive the vestibular, lateral line and auditory efferents have shown an absence of electroreceptions [193,220].

In summary, hair cells evolved from single Choanoflagellate to evolve into *Atoh1* dependent hair cells of vertebrates. Mechanoreception depends on polarity for the inner ear and lateral line, which may counteract of some vestibular and lateral line hair cells or organize unipolarity of canal cristae and most auditory hair cells. Tip links form between stereocilia to open the channel depending on the evolution of *Tmc1/2*. Electroreception does not evolve into mechanotransduction and has no polarity, comparable to Choanoflagellates.

## 5. Conclusions

Choanoflagellates are the basis of animals that evolved approximately 800 million years ago. Apical kinocilia surrounded by microvilli resemble the electroreceptor hair cells, having either a central kinocilium or microvilli [22,23,27]. In contrast, the lateral line, vestibular and cochlear hair cells develop a polarity for a mechanosensory transduction channel for its function [37,44]. *Tmc1* and/or *Tmc2* are an essential connection of mechanotransduction [42], which can be traced to Choanoflagellates [43]. Further work is needed to understand all the functions of various *Tmc* forms. For example, the sequence of mechanosensory hair cells is likely expressed by *Tmc1/2*, which is unique in cyclostomes and splits into two *Tmc* genes in gnathostomes.

**Figure 6.**
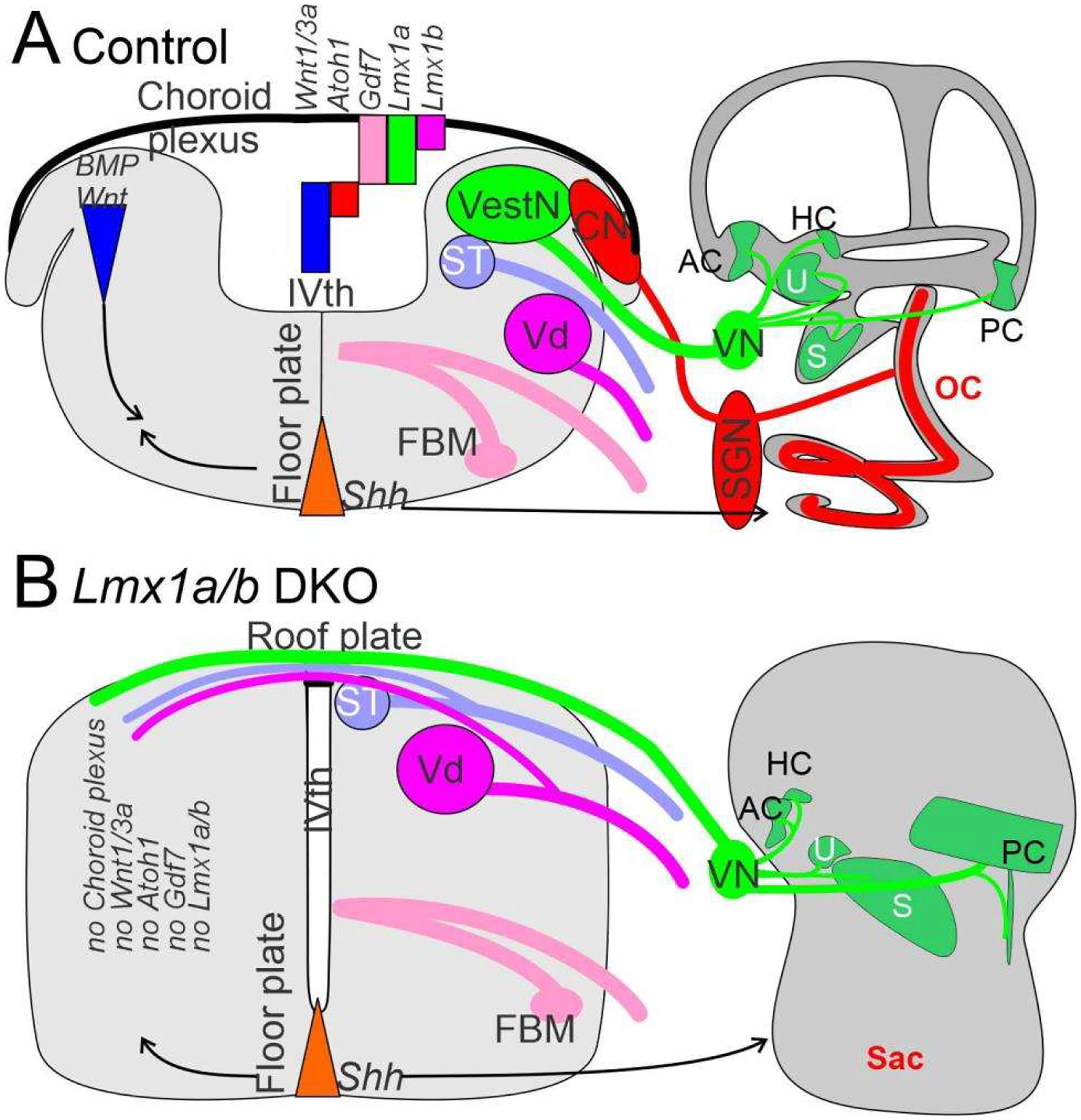
Central projections of the ear depend on the brainstem. Vestibular neurons project dorsally in the hindbrain in control and *Lmx1a/b* DKO mice (VUI; **A**,**B**). In *Lmx1a/b* DKO mice, central cochlear projections never develop as they do in controls (**A**,**B**). In addition, in *Lmx1a/b* DKO mice, vestibular projections interconnect across the roof plate, whereas vestibular fibers are normally separated by the choroid plexus (**A**,**B**). In addition to the loss of the cochlea and spiral ganglion neurons, the cochlear nucleus does not form in *Lmx1a/b* DKO mice (**B**). Furthermore, in *Lmx1a/b* DKO mice, *Atoh1*, *Gdf7* and *Wnt1/3a* expressions are absent (**A**,**B**). The signal of *Shh* drives both the ventral brainstem and ventral cochlea (arrows), which are altered without dorsal interaction and lack cochlear neurons in *Lmx1a/b* DKO mice (**A**,**B**). AC, HC, PC, anterior, horizontal, posterior cristae; CN, cochlear nuclei; FBM, facial branchial motoneurons; S, saccule; SGN, spiral ganglion neurons; ST, solitary tact; U, utricle; Vd, trigeminal; VestN, vestibular nuclei; VN, vestibular neurons. Modified after [11,28,153].

The lateral line, ear and electroreception differentiate into hair cells (*Atoh1*) that innervate vestibular neurons (*Neurog1*). In contrast to a simple critical dependency (*Atoh1* define hair cells, *Neurog1* define neurons), centrally nuclei of the brainstem depend on *Atoh1* (LL, ELL, replaced by auditory nuclei in amniotes [26]), *Neurog1/2*, *Olig3*, *Ascl1* and *Ptf1a* (VN; [25]). For the brainstem, *Shh* diffuses from ventral floor plate (Figure 6), whereas the dorsal aspect of the roof plate/choroid plexus depends on *Lmx1a/b*, *BMPs* and *Wnts* [11,28,153]. In the absence of *Lmx1a/b*, the dorsal formation does not form into a choroid plexus and lacks central nuclei, including *Atoh1* (Figure 6). The reduction of *Shh* and *Gli* may depend on the feedback between the dorsal and ventral interaction with *Lmx1a/b*. A similar interaction between *Shh* defines the cochlear hair cells [173], which interact with *Pax2*, *Lmx1a/b*, *Sox2* and *Gata3* [16,28,58,59] to eliminate cochlear hair cells, suggesting a unique interaction between *Shh* and cochlear development [11,78]. Interestingly enough, the partial formation of some vestibular hair cells in *Shh*, *Pax2*, *Lmx1a/b* and *Gata3* with a near-normal development for central vestibular nuclei (Figure 6) are downstream of *Eya1* [15].

Obviously, there is a formation of the lateral line and electroreception in most vertebrates, whereas amniotes lose the two sensory neurons, brainstem and hair cells, instead evolving an auditory system [26,122]. *Lmx1a/b* null mice showed a loss of cochlear hair cells, cochlear neurons and cochlear nuclei (Figure 6). Unfortunately, the expression of *Lmx1a/b* is required for the dorsal part of the hindbrain, which has not been analyzed in the lateral line and electroreception in gnathostomes. It is possible that the lateral line and electroreception may play a role in *Lmx1a/b* expression to help the transformation of amniotes after the loss of peripheral hair cells and associated nuclei and central projections. Recent evidence has shown that cyclostomes have a different organization of *Lmx* [153], but their expression of *Lmx1a/b* is unclear. Moreover, the two groups of teleosts that have evolved an electroreception have a unique expansion among all gnathostomes [27,135]. This expansion mimics the auditory system of amniotes [26], for which information on *Lmx1a/b* expression is lacking.
